# Decision-making about complementary and alternative medicine by cancer patients: integrative literature review

**Published:** 2014-04-15

**Authors:** Laura Weeks, Lynda G Balneaves, Charlotte Paterson, Marja Verhoef

**Affiliations:** Laura Weeks, PhD, is a Senior Research Fellow at the Ottawa Integrative Cancer Centre, Ottawa, Ontario.; Lynda G. Balneaves, RN, PhD, is an Associate Professor in the School of Nursing, University of British Columbia, Vancouver, British Columbia.; Charlotte Paterson, MSc, PhD, MBChB, MRCGP, is a Research Fellow in the School of Social and Community Medicine at the University of Bristol, Bristol, England.; Marja Verhoef, PhD, is a Professor and Canada Research Chair in Complementary Medicine in the Department of Community Health, University of Calgary, Calgary, Alberta.

## Abstract

**Background:**

Patients with cancer consistently report conflict and anxiety when making decisions about complementary and alternative medicine (CAM) treatment. To design evidence-informed decision-support strategies, a better understanding is needed of how the decision-making process unfolds for these patients during their experience with cancer. We undertook this study to review the research literature regarding CAM-related decisionmaking by patients with cancer within the context of treatment, survivorship, and palliation. We also aimed to summarize emergent concepts within a preliminary conceptual framework.

**Methods:**

We conducted an integrative literature review, searching 12 electronic databases for articles published in English that described studies of the process, context, or outcomes of CAM-related decision-making. We summarized descriptive data using frequencies and used a descriptive constant comparative method to analyze statements about original qualitative results, with the goal of identifying distinct concepts pertaining to CAM-related decision-making by patients with cancer and the relationships among these concepts.

**Results:**

Of 425 articles initially identified, 35 met our inclusion criteria. Seven unique concepts related to CAM and cancer decision-making emerged: decision-making phases, information-seeking and evaluation, decision-making roles, beliefs, contextual factors, decision-making outcomes, and the relationship between CAM and conventional medical decision-making. CAM decision-making begins with the diagnosis of cancer and encompasses 3 distinct phases (early, mid, and late), each marked by unique aims for CAM treatment and distinct patterns of informationseeking and evaluation. Phase transitions correspond to changes in health status or other milestones within the cancer trajectory. An emergent conceptual framework illustrating relationships among the 7 central concepts is presented.

**Interpretation:**

CAM-related decision-making by patients with cancer occurs as a nonlinear, complex, dynamic process. The conceptual framework presented here identifies influential factors within that process, as well as patients' unique needs during different phases. The framework can guide the development and evaluation of theorybased decision-support programs that are responsive to patients' beliefs and preferences.

It is well established that at least half of all patients with cancer use some form of complementary and alternative medicine (CAM) , such as acupuncture, massage, and natural health products, as part of their cancer care.^1^–^5^ Many factors contribute to the high prevalence of CAM use, including an increasing amount of high-quality research evidence, increased regulation and availability of natural health products, improved regulation of qualified practitioners, and cultural trends that privilege more "natural" therapies and individual involvement in self-care.^6^–^8^

Although CAM use has become common within cancer care, it remains controversial. Many CAM practices originate within philosophical traditions that deviate from Western medicine, leading some individuals to view them skeptically.^9^ Furthermore, the body of research evidence for most CAM therapies tends to be smaller and often of lower quality than the evidence for conventional medical therapies.^10^,^11^ Existing CAM research evidence is also often difficult to find, synthesize, and share with appropriate knowledge users.^12^,^13^ Finally, the potential for interactions with conventional cancer therapies is another common concern.^14^,^15^

The controversies surrounding CAM use contribute to increased levels of conflict and anxiety for patients who contemplate using these therapies as part of their cancer care.^12^,^16^ For this reason, researchers have begun to explore how and in what context patients with cancer make decisions about CAM use, primarily in an effort to design supportive interventions. Many different perspectives have been explored, including those of people with a range of cancer types,^16^–^18^ those who have declined standard care,^19^,^20^ and those who identify with a specific ethnic group.^21^,^22^ It has become clear that CAM-related decision-making by patients with cancer (hereafter referred to as "CAM and cancer decision-making") is a complex, dynamic, nonlinear, and highly individualized process. To design evidenceinformed decision-support strategies, a better understanding is needed not only of how the decisionmaking process related to use of CAM unfolds during the cancer trajectory but also of the relevant concepts and relationships.

The purpose of this study was to review the research literature regarding CAM-related decision-making by patients with cancer within the context of treatment, survivorship, and palliation. Specifically, we were interested in the process, context, and outcomes of CAM decision-making and how this decision-making process relates to that associated with conventional medical treatments. We aimed to summarize the literature, to synthesize its critical elements into a preliminary conceptual framework, and to make recommendations for future research.

## Methods

We conducted an integrative literature review^23^ of English- language research articles published since 1998 that describe CAM decision-making related to cancer treatment, survivorship, or palliation. Integrative literature reviews follow many of the same methods as systematic reviews, but their scope is broader. The intent is to synthesize a broad range of literature on an emerging topic with the goal of developing an initial or preliminary model or framework.^24^ Through this review, we intended to propose a more comprehensive, holistic understanding of CAM and cancer decision-making than has been possible through any primary research study.

Our search strategy was developed with the assistance of a health librarian and included both subject headings and keywords related to cancer, decision-making, and CAM or integrative medicine (online Appendix A). We searched the following electronic databases through September 2011: Academic OneFile, Alt HealthWatch, Allied and Complementary Medicine Database, CINAHL, EBSCO, Embase, MEDLINE, OmniFile, PsycINFO, PubMed, SocINDEX, and Sociological Abstracts. We included articles that described either or both of (1) the process or context of CAM decision-making relevant to cancer treatment, survivorship, or palliation; and (2) the outcomes of the decision-making process. We defined a "process" as a series of actions, changes, or reactions that happen over time as an individual contemplates CAM treatment options. We defined "context" as the set of circumstances within which decision-making takes place. We defined "outcomes" as the results of the decision-making process (and not of the cancer). Pertinent articles were included whether CAM decision-making was considered as a separate issue or as an issue alongside conventional medical decision-making. We excluded articles that described decision-making related to cancer prevention and those that focused exclusively on CAM use or the context of CAM use, although (as stated above) we included articles that described the context of CAM decision-making. All of the authors participated in the screening process, with various pairs of authors independently screening each article title and abstract for eligibility. For articles where it was difficult to determine eligibility on the basis of title and abstract alone, the full text of the article was retrieved and examined before eligibility was determined. Screening decisions were recorded in an Excel database and were compared by one reviewer (L.W.) for consistency. Discrepancies were discussed and resolved during a team teleconference during which all reviewers had access to all abstracts and/or full-text articles as required. Once a preliminary list of included articles had been developed, we reviewed the reference list of each article for other potentially eligible articles missed in the initial search.

Descriptive data and results of the included studies were extracted from each article by one reviewer (L.W.). Descriptive data included such items as first author, article title, research purpose, sample size, and study design. In addition, the reviewer extracted verbatim result statements from each of the included studies. A quality assessment was not conducted, as such an assessment is outside the scope of an integrative literature review. Descriptive data were analyzed by calculating frequencies for relevant categories within each variable. Result statements were analyzed through an iterative process, with the goal of identifying distinct concepts relevant to CAM decision-making. The reviewer began by reading each article to ensure a comprehensive understanding of the content. Next, the reviewer extracted individual result statements and grouped them within Altas.ti qualitative software according to unique concepts within CAM decisionmaking that the statements represented. As each result statement was extracted, it was compared with all previously extracted statements, so that it could be grouped with similar statements or placed into a new category. Each category was labelled with a term to describe a concept within CAM decision-making that the statements in the category represented. Labels were emergent, based on careful reading of the data and regular team discussions, and were not necessarily borrowed from the reviewed articles, to avoid privileging any particular perspective from the existing literature. In the course of this analysis, a set of 7 unique analytic categories emerged. The final step was to synthesize the result statements within each category and propose a preliminary conceptual framework. One reviewer conducted the majority of the analysis; however, regular teleconferences and email discussions with the research team helped to confer authenticity within the emerging analytic categories.

The predefined review protocol is available from the corresponding author upon request.

## Results

**Figure 1:**
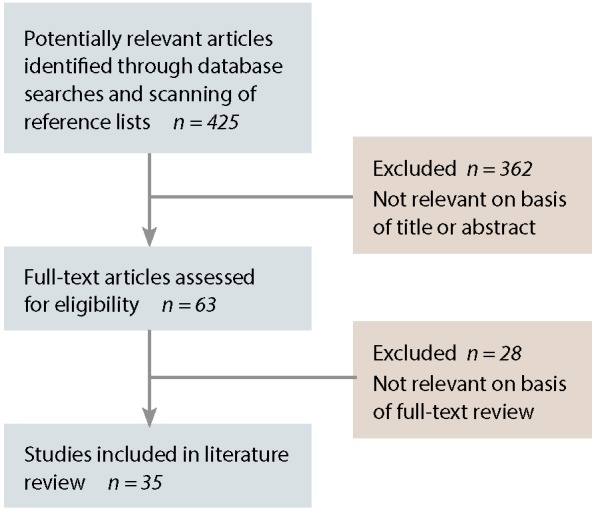
Results of search strategy and process of identifying articles related to complementary and alternative medicine and decision-making by patients with cancer.

We identified 425 articles by searching the electronic databases and scanning reference lists. Of these, 35 articles^6^,^12^,^17^–^22^,^25^–^51^ met our criteria and were included in the review (Figure 1).

### Descriptive analysis

Over half of the included articles (19 [54%]) described studies that had been conducted in Canada. The majority (31 [89%]) included participants with any stage of cancer, and over half (19 [54%]) described studies that focused on all cancer types. One-third (12 [34%]) of the included articles described studies that explored CAM and cancer decision-making from the perspective of one or more special populations: various ethnic groups (6 [17%]), those who declined some form of conventional treatment (5 [14%]), significant others (1 [3%]), and participants in phase I clinical trials (1 [3%]). The majority of these articles (25 [71%]) described qualitative research studies, whereas the others reported on cross-sectional surveys (5 [14%]), mixed-methods studies (3 [9%]), or synthesis research (2 [6%]). Table 1 summarizes the descriptive results.

**Table 1:** Descriptive information about 35 articles describing decision-making by patients with cancer

Reference	Purpose	Use of theory	Cancer type, special population	Country	Method or type of analysis	Sample size
Balneaves et al.^12^	To explore the personal and social processes engaged in by women with early-stage breast cancer when making decisions about CAM during the period from diagnosis to survivorship	Emergent, developed by authors ("Bridging the Gap")	Breast (stage I and II)	Canada	Grounded theory	20
Balneaves et al.^25^	To provide a preliminary description of use of complementary therapies by women living with breast cancer and the predisposing factors associated with the decision to use complementary therapies	Not stated	Breast	Canada	Cross-sectional survey	64
Bishop and Yardley^26^	To explore the positioning of people within accounts of treatment decisions and thereby to explicate strategies used to manage ideological conflict within the context of orthodox and complementary medicine in cancer	Positioning theory	All	United Kingdom	Discourse analysis	43
Boon et al.^27^	To investigate the question, "Are users of CAM more autonomous than non-users with respect to problem-solving and decision-making preferences?"	Deber–Kraestchmer problem-solving decision-making scale	All	Canada	Cross-sectional survey	489
Boon et al.^28^	To explore prostate cancer patients' perceptions, feelings, ideas, and experiences regarding making decisions to use or not use CAM	Push–pull factors	Prostate	Canada	Content analysis	29
Boon et al.^29^	To examine breast cancer patients' perceptions of, approaches to, and experiences with CAM	Push–pull factors	Breast	Canada	Content analysis	36
Brazier et al.^30^	To evaluate the impact of participating in an integrative cancer care program at the Centre for Integrated Healing in Vancouver, British Columbia, on patients' lifestyle, quality of life, and overall well-being	Not stated	All	Canada	Interpretive description	28
Broom^31^	To understand how individuals with cancer make decisions about the legitimacy of ideas, expertise, treatments, and regimens in the context of their cancer and to develop a conceptualization of therapeutic decision-making, using the notion of bricolage* as a key point of departure	Not stated	All; intensive CAM users	Australia	Interpretive qualitative research	20
Broom and Tovey^32^	To examine individual cancer patients' temporal experiences of CAM, including (1) the disciplining of the self demanded by certain CAM therapeutics and the impact of that on the experience of having cancer, (2) the role of CAM healing therapists in reconceptualizing disease and fi lling perceived gaps in biomedical cancer care, and (3) the complex interplay between CAM-derived notions, self-healing, and the state of nearing death	Not stated	All; intensive CAM users	United Kingdom	Interpretive qualitative research	8
Broom and Tovey^33^	To examine cancer patients' perspectives on the nature of evidence and the degree to which different understandings of evidence inform decision-making about CAM and biomedicine	Social theory (postmodernity, reflexivity, technologies of the self, dialectic)	All	Canada	Interpretive qualitative research	80
Brown et al.^34^	To elucidate common themes across 3 studies of women's decisionmaking and to examine the process that women undergo in making an important decision about their health and well-being, including where and how they acquire the necessary information to make a decision, the factors that influence their decision, who supports them in the decision-making process, and how they reconcile confusing or conflicting information	Emergent, developed by authors	Breast	Canada	Constant comparison	36
Chiu et al.^35^	To explore, in a sample of Chinese patients with cancer, (1) the general conceptualization of CAM use, (2) the meaning of CAM use in relation to cancer, (3) the patterns of CAM use before and after cancer diagnosis, (4) the reasons for CAM use, and (5) the sociocultural process in making decisions about CAM use	Emergent, developed by authors	All types (stages I, II, and III); Chinese patients	Canada	Constant comparison	14
Evans et al.^36^	To explore the processes shaping men's decision-making about CAM and the rationales they provide for their views and behaviour	Not stated	Male cancer, any type	United Kingdom	Constant comparison	34
Evans et al.^37^	To explore the use and evaluation of CAM-related information by male cancer patients	Not stated	Male cancer, any type	United Kingdom	Not specific; thematic	34
Gray et al.^38^	To explore cancer patients' motivations for seeking information about unconventional therapies, their decision-making processes, their experiences with such therapies, their attempts to communicate with conventional health care practitioners, and their perceptions of family members and friends' reactions to their interest in unconventional therapies	Not stated	All	Canada	Not specific; thematic	32
Hlubocky et al.^39^	To describe the general usage rates of biologically based CAM among participants in phase I trials; secondary objectives were to explore social and demographic factors associated with CAM use, to describe potential differences in treatment decision-making preferences among CAM users and non-users, and to investigate associations of CAM use with awareness of prognosis and quality of life	Not stated	Advanced cancer; phase I trial participants	United States	Cross-sectional survey	212
Jones et al.^22^	To explore the beliefs and attitudes of African American survivors of prostate cancer regarding the use of CAM	Not stated	Prostate; African American patients	United States	Cross-sectional survey and phenomenology	14
Kakai et al.^40^	To investigate ethnic differences in health information–seeking behaviours among cancer patients of diverse ethnicity in Hawaii; also, to explore a possible association between patients' education and ethnicity and choice of health information	Not stated	All; various ethnic groups	United States	Correspondence analysis	140
Kimby et al.^41^	To examine the relationships between user profiles (sociodemographic factors, treatment orientations, cancer status) and users' choice of various unconventional types of treatment (individualized versus standardized unconventional treatments)	Not stated	All	Denmark	Cross-sectional survey	441
Markovic et al.^17^	To explore the impact of specific social and cultural factors influencing health care decision-making	Not stated	Gynecologic	Australia	Grounded theory	53
Montbriand^42^	To recreate a model reflecting the health decision realities of patients with a diagnosis of cancer of the respiratory or digestive system	Naturalistic and rationalistic research; phenomenology; heuristics; Tversky's elimination-by-aspects theory; and emergent, developed by authors	Respiratory and digestive	Canada	Ethnography (following phenomenology)	300
Oh and Park^43^	To explore how patients with cancer choose a therapy after the diagnosis has been made and the decision-making strategies used by these patients when they visit a doctor or when they use alternative therapies	Not stated	All	Korea	Cognitive ethnographic decision tree model	194
Ohlén et al.^44^	To explore how significant others were involved in cancer patients' decision-making processes related to CAM	Not stated	Breast and prostate (early and advanced); significant others	Canada	Grounded theory	40 with early cancer; 21 with advanced cancer; 31 significant others
Owens^21^	To describe the self-help theoretical framework in relation to CAM and to delineate relationships in Braden's Self-Help Model of sideeffect burden to uncertainty, CAM self-care, and quality of life in Hispanic women undergoing treatment for breast cancer	Braden's Self-Help Theory	Breast; Hispanic patients	United States	Cross-sectional survey	144
Ritvo et al.^45^	To apply a theoretical model, the Risk Adaptation Model, to further the clinical understanding of the motivations of cancer patients in seeking complementary therapies	Risk Adaptation Model	All	Not original research	Not original research	Not original research
Shumay et al.^20^	To examine cancer patients' reasons for declining all or part of recommended cancer treatment and choosing CAM	Montbriand's decisiontree model	All; various ethnic groups; patients who declined conventional treatment	United States	Thematic	14
Singh et al.^6^	To compare the perceptions, beliefs, ideas, and experiences that contribute to the decision of patients with prostate cancer to use or not to use CAM	Not stated	Prostate; various ethnic groups	United States	Thematic	27
Truant and Bottorff 18	To examine the decision-making process for complementary therapies from the perspective of women with breast cancer in the context of the cancer trajectory	Emergent, developed by authors	Breast	Canada	Grounded theory	16
Verhoef et al.^46^	To describe the type of information about CAM that patients with cancer use in their decision-making, to understand why certain types of information about CAM are accepted as evidence by patients with cancer, and to explore the role of scientific evidence in treatment decision-making	Not stated	All	Canada	Content analysis	27
Verhoef et al.^47^	To summarize and review the reasons for CAM use, as well as the sociodemographic and disease characteristics associated with CAM use among patients with cancer	Not stated	All (focus on breast and prostate)	United States, Canada, Western Europe, Asia, Middle East, Australia, New Zealand	Systematic review	52 articles
Verhoef and White^19^	To explore why and how patients with cancer decide to forgo conventional treatments in favour of alternative treatments, as well as which factors influence this decision	Not stated	All; patients who declined conventional treatment	Canada	Content analysis	31
Verhoef et al.^48^	To explore cancer patients' experiences with and expectations of the role of family physicians in discussing complementary therapies	Not stated	All	Canada	Content analysis	14
White et al.^49^	To explore why men decline conventional prostate cancer treatment and use CAM instead, to understand the role of holistic healing in their care, and to document their recommendations for health care providers	Not stated	Prostate; patients who declined conventional treatment	Canada	Content analysis	29
White and Verhoef 50	To explore the role of spirituality in cancer management and decisionmaking for men with prostate cancer who declined conventional treatment	Not stated	Prostate; patients who declined conventional treatment	Canada	Thematic	10
White and Verhoef 51	To explore in depth how sense of control was related to the decision to forgo conventional treatment for prostate cancer and to use CAM therapies for cancer	Not stated	Prostate; patients who declined conventional treatment	Canada	Content analysis	8

CAM = complementary and alternative medicine.

* Bricolage was described in this paper as the active process in which people engage to construct their unique understanding of CAM, by piecing together ideas that fit with their needs and experiences from diverse practices and models of care.

### Emerging concepts in the CAM and cancer decision-making literature

Seven unique concepts related to CAM and cancer decision-making emerged through our analysis: decision- making phases, information-seeking and evaluation, decision-making roles, beliefs, contextual factors, decision-making outcomes, and the relationship between CAM and conventional medical decision-making. Table 2 provides a guide to which articles included data relevant to each concept, and the results for each category are briefly synthesized below.

**Table 2:** Concepts related to decision-making in the context of complementary and alternative medicine and cancer described in articles included in the review

Reference	Decision-making phases	Information-seeking and evaluation	Decision-making roles	Beliefs	Contextual factors	Decision-making outcomes	Relationship between CAM and conventional medical decision-making
Balneaves et al.^12^	×	×	×	×	×	×	×
Balneaves et al.^25^			×	×			
Bishop and Yardley^26^			×		×	×	
Boon et al.^27^			×				
Boon et al.^28^	×	×		×	×	×	×
Boon et al.^29^	×	×	×	×	×	×	
Brazier et al.^30^		×			×	×	
Broom^31^	×				×		
Broom and Tovey^32^				×	×	×	
Broom and Tovey^33^	×	×		×	×	×	×
Brown et al.^34^	×	×		×	×	×	×
Chiu et al.^35^	×	×		×	×	×	
Evans et al.^36^	×	×	×	×	×	×	
Evans et al.^37^	×	×		×	×	×	×
Gray et al.^38^	×	×		×	×	×	
Hlubocky et al.^39^			×				
Jones et al.^22^		×		×	×	×	
Kakai et al.^40^		×		×	×		
Kimby et al.^41^					×	×	
Markovic et al.^17^		×		×	×	×	
Montbriand^42^	×	×	×	×	×	×	
Oh and Park^43^		×		×	×		
Ohlen et al.^44^	×		×		×	×	
Owens^21^					×	×	
Ritvo et al.^45^	×	×	×	×		×	
Shumay et al.^20^		×		×			
Singh et al.^6^				×	×	×	×
Truant and Bottorff 18	×	×		×	×	×	×
Verhoef et al.^46^	×	×	×	×	×	×	×
Verhoef et al.^47^					×	×	
Verhoef and White^19^				×	×	×	
Verhoef et al.^48^			×	×	×		
White et al.^49^	×	×	×	×	×	×	
White and Verhoef 50		×		×	×	×	
White and Verhoef 51		×	×	×	×	×	

### Decision-making phases

The studies included in our review illustrate that CAM-related decision-making does not happen at any finite point in time but rather occurs as a nonlinear, complex, dynamic process, of which therapy choices are one outcome.^18^,^29^,^33^ Although each person follows his or her own unique CAM decision-making process, we identified 3 specific phases (which we labelled early, mid, and late) that correspond to different events across the cancer trajectory, involving different aims and patterns of information- seeking and evaluation.^42^

The early phase of CAM decision-making begins with the initial diagnosis or a recurrence of cancer.^18^,^28^,^37^ It is characterized by feelings of fear and a sense of loss of control.^28^ A wide range of CAM therapies are typically contemplated^12^ during this phase, and the process involves seeking and evaluating information regarding the pros and cons of each therapy and reaching a decision regarding whether or not to use CAM, and if so, which type.^12^,^29^,^34^,^35^ Some people seem to move through this phase quickly and to spend little time, if any, researching CAM options.^18^ Those with past CAM experience seem to fall into this category, as they tend to be less overwhelmed with the amount of available and conflicting information.^18^,^46^ Others spend more time consulting a range of information sources to help evaluate the potential of CAM use.^29^

The mid phase is best viewed as a maintenance phase, with the aim being to develop a personalized regimen of CAM therapies that fits within the individual's beliefs and needs. Patients seem to transition to this phase of decision-making when they encounter some sort of positive change in their personal context, for example, once they have adapted psychologically to their cancer diagnosis or completed their conventional cancer treatment. CAM therapies used during this phase are directed toward maintaining well-being, controlling the spread of cancer cells, managing the side effects of treatment, boosting the immune system, and preventing or delaying recurrence.^18^,^47^

The late phase of decision-making includes the same iterative information-gathering and evaluation apparent during the early phase, but there is less urgency, a stronger awareness of CAM, and more comfort with a variety of information sources.^12^ People seem to transition to this late phase when their conventional treatment ends and they move into survivorship or palliative care.^12^,^37^ During the late phase, patients consider CAM therapies that help to address a variety of aims, including overcoming a sense of loss and abandonment after discharge, maintaining health, prolonging life, or coming to terms with impending death.^37^ In palliative situations, the patient may re-evaluate CAM regimens that were previously perceived to require too much time, money, and effort.^32^

Transitioning between phases seems to correspond to times of crisis or change within the cancer experience^42^ that modify perceived consequences or expectations of CAM therapies within cancer treatment.^45^ Such changes seem to motivate people to revisit their original CAM-related decisions and to renew the process of gathering and evaluating information to help adapt to a new circumstance.^12^,^18^,^37^,^38^ This transitioning does not appear to represent desperation on the part of patients but, instead, a reasoned approach to critically examining their situation and available options.^33^

### Information-seeking and evaluation

Information-seeking and evaluation are integral components of decision-making, with distinct patterns during each phase. For some people, these activities form a process that begins at diagnosis and continues throughout their cancer journey. Other people begin to seek and evaluate information when they transition between decision-making phases and need to revisit their CAM decisions. People tend to rely on a wide range of information sources, including books, the Internet, mass media, CAM and conventional practitioners, friends and family, and other cancer patients.^12^,^17^,^18^,^20^,^29^,^35^,^36^,^37^,^49^,^51^ Preferred information sources differ depending on the decisionmaking phase, with the broadest range of information sources used in early-phase decision-making, when individuals are exploring their treatment options and learning what types of CAM are available. In subsequent phases, individuals tend to rely on personal experience and the results of medical tests to evaluate whether CAM is helping them to achieve their treatment goals.^20^,^35^,^49^,^50^,^51^

The process of evaluating information has largely been studied by examining the meaning of evidence when cancer patients make CAM-related decisions. It is clear from our review of this literature that what constitutes high-quality evidence for the safety and effectiveness of CAM varies greatly among individuals^46^ and also diverges from the standard applied within evidence-based medicine.^12^,^36^,^45^,^51^ The type and source of information that individuals accept as evidence seem to depend mostly on underlying beliefs and values, perceived credibility of information, experience with CAM, and stage of disease.^18^,^29^,^31^,^36^–^38^,^46^ Anxiety, ethnicity, and social support may also play a role.^12^,^18^,^40^ Within CAM decision-making, information evaluation will play a stronger or weaker role, depending on the level of attention that the individual affords to a given content area and his or her beliefs regarding the potential for use of CAM to modify his or her condition.^45^ For example, if someone feels strongly that using herbal medicine can help mitigate the side effects of cancer treatment, and side effects are a great concern for that person, information evaluation becomes an important part of the decision-making process; however, if side effects are not as important an issue for the patient, then information evaluation in this situation is less important.

### Decision-making roles

Individuals tend to take either an active or a passive role in decision-making, and the role they choose may differ at different points during the decision-making process.^12^,^26^,^29^,^36^,^39^,^42^,^45^ People who take an active role appear more self-motivated,^42^ have more self-confidence,^12^ and are more likely to have used CAM before their cancer diagnosis^36^,^46^ than those who take a passive role. The more active group also embraces a wider range of CAM therapies than the more passive group.^36^ Taking an active or passive role is associated with cancer type and state of illness: those with rare forms of cancer, faster-growing tumours, or advanced disease are more likely to take an active role.^36^ Regardless of whether their role is active or passive, patients with cancer appear to experience CAM decision-making as problematic. Taking an active role often requires going against the socially sanctioned expertise of medical doctors and assuming responsibility for one's own decisions, whereas taking a passive role conflicts with the ideal of individual responsibility for health.^26^

### Beliefs

A range of beliefs influence CAM and cancer decision-making, including beliefs about the causes of cancer,^17^,^19^,^49^,^50^ treatment mechanisms,^20^,^37^,^50^ risks and benefits of CAM use,^6^,^12^,^17^,^19^,^20^,^22^,^25^,^28^,^29^,^34^,^35^,^42^,^43^,^46^,^49^–^51^ risks and benefits of conventional care,^6^,^17^–^20^,^28^,^35^, ^38^,^42^,^43^,^46^,^49^–^51^ available evidence,^18^,^19^,^31^ and disease status.^12^,^18^,^42^,^43^,^45^ A lthough it is possible to categorize beliefs in this way, it is more likely that an individual's entire belief system influences the CAM decisionmaking process, such that decisions are generally congruent with the complexity of the belief system. Depending on an individual's particular context at any given time, he or she will prioritize some beliefs over others when making decisions. For example, during active treatment, patients may prioritize their beliefs about treatment mechanisms and risks and benefits of care over their beliefs about the causes of their cancer.

It is clear, however, that not every person with cancer is explicitly aware of his or her beliefs; furthermore, these beliefs are not static. Current beliefs are informed by a range of factors, including past experiences of the individual^28^ or his or her significant others,^22^ ethnocultural values,^35^,^40^ faith in God,^22^,^42^ and education.^40^

### Contextual factors

Several contextual factors influence the experience of making CAM decisions, including demographic and disease-related factors, social factors, and cultural norms. Relevant demographic and disease-related factors include age,^33^,^47^ geography,^29^,^47^ disease status and active treatment,^12^,^28^,^33^,^38^,^46^ experience with CAM use,^19^,^28^,^35^,^38^,^49^ and income and ability to pay.^21^,^29^,^35^,^38^,^47^ Social factors centre on an individual's interactions with others, including friends and family, health care practitioners, and other patients. Finding validation and support from others appears to be of great value to patients and seems to offer them the confidence to move forward with decisions that feel right for them.^12^,^17^,^19^,^30^,^34^,^35^,^44^ In some cases, however, support and recommendations from members of a support network can result in feelings of pressure and uncertainty.^35^,^44^ Cultural norms have a strong influence on decision-making and appear to reflect a conflict between CAM and biomedicine,^12^,^33^ the limits of biomedicine,^6^,^17^,^28^,^29^,^35^–^37^ and perceived harmlessness of many CAM therapies.^28^,^29^

### Decision-making outcomes

The CAM decision-making process contributes to a range of outcomes, including the decision to use or not use CAM, but also several others. The process of making a decision has been documented to empower individuals through more active participation in their own care,^49^ which can increase a person's sense of control and thus reduce anxiety and fear.^12^,^18^,^19^,^30^,^37^,^42^,^44^,^50^,^51^ CAM decision-making also introduces individuals to different philosophies of healing, healthy lifestyle behaviours, and personal development.^46^ However, making decisions about CAM may also be associated with certain difficulties. For example, common outcomes of CAM decision-making include conflict and resistance from clinicians, both of which can contribute to feelings of frustration and anxiety about making the "right" decision.^12^,^30^,^31^ Furthermore, individuals may describe feeling uncomfortable with the added responsibility and self-accountability that use of CAM can bring.^26^,^32^,^34^

### Relationship between CAM and conventional medical decision-making

Making decisions about CAM cannot be separated from making decisions about conventional medicine.^6^,^28^,^34^ These may seem to be similar processes that occur concurrently^46^; however, depending on the situation, one or the other will take priority.^18^ Furthermore, the goals of both decision- making processes appear to be the same, but an individual's beliefs and values will lead to a choice of either CAM or conventional treatment (or both) to achieve his or her treatment goals.^6^

## Synthesis

**Figure 2:**
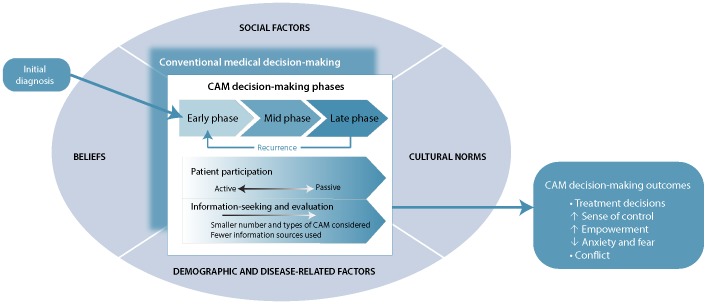
Conceptual framework of the decision-making process for complementary and alternative medicine (CAM) by patients with cancer. Conventional medical-decision making is included in this framework because making decisions about CAM cannot be separated from making decisions about conventional medicine. Social factors, cultural norms, and demographic and disease-related factors constitute the "contextual factors" discussed in the text. Transitions from one phase to another within the decision-making trajectory may occur at times of crisis or milestones, such as the end of conventional treatment and transition to survivorship or palliative care.

An emergent conceptual framework illustrating the relationship among the 7 central concepts is presented in Figure 2. In this framework, CAM decisionmaking begins with the diagnosis of cancer. The process encompasses 3 distinct phases, each marked by unique patterns of information-seeking and evaluation, specifically, early-, mid-, and late-phase decision-making. Transitions between phases correspond to changes in health status, a crisis, or other milestones within the cancer trajectory. All decision-making phases are influenced by a myriad of contextual factors, including demographic and disease-related factors, social factors, cultural norms, and personal beliefs about cancer, its causes, and its treatments. Outcomes of the decision-making process include one or possibly multiple CAM decisions over time, and also shifts in perceived sense of control, empowerment, anxiety, and fear, as well as conflict over whether the "right" decision has been made.

## Interpretation

Through this integrative literature review, we have created a conceptual framework for CAM and cancer decision-making that can be used to guide the development of decision-support programs as well as future research in this field. The inclusion of diverse studies representing diverse populations ensures that the framework is comprehensive and therefore broadly applicable to cancer patients who are contemplating treatment options. It illustrates 3 distinct phases within CAM and cancer decision-making, each characterized by different patterns of information-seeking and evaluation. It is also clear that CAM decision-making should not be considered as a process separate from decision-making related to conventional medical care. Beliefs, values, and other social and cultural norms guide all treatment choices, and some patients will require support to articulate and prioritize these factors when making treatment decisions.

The inclusion of diverse study designs within integrative literature reviews means that such reviews are more susceptible to lack of rigour than are other types of reviews (such as systematic reviews).^24^ For example, although our search was extensive, it is possible that we missed some primary studies, especially any published in languages other than English. However, given that we searched multiple databases and scanned reference lists of included articles for additional eligible studies, it is likely that we identified most of the published literature in this field. Furthermore, the reliability of our sampling strategy was enhanced by using pre- specified inclusion and exclusion criteria and by having 2 reviewers screen each potentially relevant article. Data extraction within integrative literature reviews can be especially problematic because of the wide range of variables, theories, and populations examined within diverse primary studies. To provide focus and delineate boundaries for the review, our team met frequently to formulate a clear research purpose and related data-extraction strategy, as well as to discuss the analysis as it was emerging.^24^ Finally, our data analysis strategy was compatible with strategies used to combine diverse data within mixed-methods studies,^52^ further supporting the rigour of our review.

We expect that the results of this review, including the conceptual framework and descriptions of relevant concepts within CAM and cancer decision-making, will be instructive for health care professionals who are supporting patients moving through this complex process. Decisions about CAM use are often characterized by conflict and anxiety,^12^,^16^ perhaps more so in the setting of cancer than other diseases, given the cultural significance of a cancer diagnosis.^53^ Decision-support programs are needed to promote open dialogue about the use of CAM in cancer care, to direct patients to high-quality information resources, and to support the safe integration of CAM within standard care.^54^ This study has helped to identify some key characteristics required of such decision-support programs. Of note, these programs should encompass a variety of strategies to support patients within different decisionmaking phases. They must also acknowledge the variability and complexity of individuals' personal contexts, including beliefs, values, and social roles, which will influence when and how people make treatment decisions. Decision-support programs must also be flexible and adaptive, to account for both active and passive decision-making roles, diversity in preferred information sources, and changing needs and goals throughout the cancer experience. Finally, given that CAM-related decisions are intertwined with decision-making related to conventional medical treatments, it seems reasonable that CAM decision-support programs should be integrated with other programs offered within standard care.

To date, most of the research in the field of CAM and cancer decision-making has been conceptual and exploratory. This perspective has been crucial to gaining a better understanding of the complexity within CAM decision-making. The integrative review presented here provides a comprehensive understanding of the CAM and cancer decision-making process, including the distinct decision-making phases, roles, and contextual influences. It is now time to move forward with the development and evaluation of theory-based decision-support programs to provide evidenceinformed support for cancer patients in making decisions about CAM and conventional medical treatment. The proposed conceptual framework is a guide to ensure that decision-support programs are responsive to patients' beliefs and preferences and appropriate to their unique needs at different points throughout the cancer trajectory.
